# *Cannabis*, the Endocannabinoid System and Immunity—the Journey from the Bedside to the Bench and Back

**DOI:** 10.3390/ijms21124448

**Published:** 2020-06-23

**Authors:** Osnat Almogi-Hazan, Reuven Or

**Affiliations:** Laboratory of Immunotherapy and Bone Marrow Transplantation, Hadassah Medical Center, The Faculty of Medicine, Hebrew University of Jerusalem, Jerusalem 91120, Israel; reuvenor@hadassah.org.il

**Keywords:** *Cannabis*, cannabinoid, immune, inflammation, infectious diseases, virus, bacteria, cancer, autoimmune, transplantation

## Abstract

The *Cannabis* plant contains numerous components, including cannabinoids and other active molecules. The phyto-cannabinoid activity is mediated by the endocannabinoid system. Cannabinoids affect the nervous system and play significant roles in the regulation of the immune system. While *Cannabis* is not yet registered as a drug, the potential of cannabinoid-based medicines for the treatment of various conditions has led many countries to authorize their clinical use. However, the data from basic and medical research dedicated to medical *Cannabis* is currently limited. A variety of pathological conditions involve dysregulation of the immune system. For example, in cancer, immune surveillance and cancer immuno-editing result in immune tolerance. On the other hand, in autoimmune diseases increased immune activity causes tissue damage. Immuno-modulating therapies can regulate the immune system and therefore the immune-regulatory properties of cannabinoids, suggest their use in the therapy of immune related disorders. In this contemporary review, we discuss the roles of the endocannabinoid system in immunity and explore the emerging data about the effects of cannabinoids on the immune response in different pathologies. In addition, we discuss the complexities of using cannabinoid-based treatments in each of these conditions.

## 1. Introduction

The medical use of *Cannabis* is several thousand years old. The first historical evidence of *Cannabis* use in traditional medicine is documented in the ancient Chinese Pharmacopoeia, written in the first century BCE [1]. In Chinese medicine, *Cannabis* was used to treat rheumatic pain, constipation, malaria, beriberi, and gynecological disorders. Evidence of *Cannabis* use in ancient traditional medicine was uncovered in Japan, India, Iran, and Egypt as well as in Arabic medicine [2]. The medical use of *Cannabis* spread to the west only around the first century CE. Across the ancient world, the *Cannabis* treatment was documented to relieve a wide range of ailments including otitis, diarrhea, asthma, arthritis, and as a topical treatment of swellings and bruises. In parallel, *Cannabis* was historically used also during religious services and for recreational purposes [2]. Its use continued worldwide until 1925, when an agreement was reached to regulate and control both the commerce and the use of opium, cocaine, and other drugs. In 1941, *Cannabis* was removed by the United States from the National Formulary and from Pharmacopeia [3].

Although it was considered a dangerous drug in many countries worldwide, during the second half of the 20th century, basic research led to the discovery of cannabinoids, cannabinoid receptors, and the endocannabinoid system [4]. In recent years, under increasing pressure from patients and their families and the emergent problem of opiate dependency, an increasing number of countries have introduced more permissive policies on the use of *Cannabis*-based medical treatments. However, the limited evidence-based data on the efficacy and side effects of different *Cannabis*-based treatments in various medical conditions, prevents many physicians from suggesting such treatments to their patients. The accumulation of data from recent studies and results from ongoing research, will hopefully allow physicians to reach a more thorough understanding of *Cannabis*-based treatments allowing the appropriate application of cannabinoids to the clinic.

## 2. The Phyto-Cannabinoid Activity Is Mediated by the Endocannabinoid System and Other Physiological Signaling Systems

*Cannabis* contains numerous molecules, including more than 60 chemical compounds classified as cannabinoids, and the different *Cannabis* chemotypes vary in their cannabinoid composition [5]. The two most extensively studied phyto-cannabinoids (natural plant cannabinoids) are D9 tetrahydrocannabinol (THC) and cannabidiol (CBD). Other active phytochemicals include Terpenes and Phenolic Compounds such as flavonoids.

The diverse and powerful effects of many phyto-cannabinoids on the human (and animal) physiology are the result of their binding with the endogenous cannabinoid receptors and are affected by the levels of their endogenous ligands. These receptors and ligands together with the enzymes and transporters which control their metabolism comprise the endocannabinoid system. This endogenous cannabinoid system is conserved throughout evolution from coelenterates to man [6].

The “classical” members of the endogenous system are the endocannabinoids *N*-arachidonoylethanolamine (anandamide, AEA) and 2-arachidonoylglycerol (2-AG), and the cannabinoid receptors: Cannabinoid receptor 1 (CB1) and Cannabinoid receptor 2 (CB2). AEA is synthesized from membrane phospholipid precursors mainly by the sequential action of *N*-acyltransferase (NAT) and Nacyl-phosphatidylethanolamines-specific phospholipase D (NAPE-PLD). 2-AG is synthesized by two diacylglycerol lipases (DAGLα/β). The endocannabinoids bio-activities are terminated by their fast catabolism, mainly through hydrolysis by a fatty acid amide hydrolase (FAAH), for AEA, and by a monoacylglycerol lipase (MAGL), for 2-AG. AEA has been demonstrated to have a high affinity for CB1 and a low CB2 affinity, while 2-AG has a moderate affinity for both receptors [7,8]. Recently, more molecules were defined as endocannabinoids including 2-arachidonoyl glyceryl ether (noladin ether, 2-AGE), *O*-arachidonoylethanolamine (virodhamine), *N*-arachidonoyldopamine (NADA), and oleic acid amide (oleamide, OA) [9].

The cannabinoid receptors CB1 and CB2 are members of the G protein-coupled receptor (GPCR) family. GPCR’s share common structural motifs, including the characteristic seven α-helical structured transmembrane domains, an extracellular N-terminus, and a changeable intracellular C-terminus [10]. CB1 and CB2 were discovered about 30 years ago [11,12,13], however, their crystal structure was only recently determined, allowing a better understanding of ligand selectivity and function [14,15]. CB1 is mainly expressed in the brain and is therefore the main mediator of the psychoactive effects of *Cannabis*, whereas CB2 has a greater expression in the immune system. CB2 expression in lymph nodes and spleen is higher than in peripheral blood cells and is different in various immune cell populations (B cells > NK cells > monocytes > neutrophils > CD8 T-cells > CD4 T-cells) [16,17]. Both receptors couple to G_i/o_ proteins and modulate various intracellular signal transduction pathways, including inhibition of adenylyl cyclase activity, calcium channels and D-type potassium channels, increase in the phosphorylation of mitogen-activated protein kinases (MAPK), and activation of A-type potassium channels. However, CB1 unlike CB2, has been reported to activate other G proteins in certain circumstances [18,19].

In addition to CB1 and CB2 receptors, endocannabinoids have also been reported to modulate several other receptors and channels including several transient receptor potential (TRP) channels, GPCRs such as GPR 55, GPR18, GPR119, γ-aminobutyric acid (GABA) A, glycine receptors, and the nuclear receptor peroxisome proliferator-activated receptor gamma (PPAR-γ) [7,20].

Endocannabinoids are implicated in numerous physiological and pathological processes. Dysregulation of the endocannabinoid system, owing to variation in the concentration of endocannabinoids or the expression and function of cannabinoid receptors and enzymes, has been associated with several conditions, such as acute stress, autism, Alzheimer’s disease, Mastocytosis, and cancer [21,22,23,24,25]. The accumulated discoveries on the endocannabinoid system triggered the search for targeted cannabinoid-based therapeutics [26].

THC and some of the other phyto-cannabinoids mediate their biological effects primarily through the classical cannabinoid receptors: CB1 and CB2. In addition, THC can act as an agonist of the receptors/channels GPR55, GPR18, PPARγ, transient TRPA1, TRPV2, TRPV3, and TRPV4, and as an antagonist of the receptors/channels TRPM8 and 5-HT3A. Moreover, THC can increase AEA and adenosine levels [8]. Interestingly enough, although CBD affects the immune function, it has a very weak affinity to CB2 or CB1, where it can act as a negative allosteric modulator [27]. Several reports have demonstrated that CBD act as an agonist of the receptors/channels TRPA1, TRPV1, TRPV2, TRPV3, PPARγ, 5-HT1A, A2 and A1 adenosine, and as an antagonist of the receptors GPR55, GPR18, and 5-HT3A. CBD is also an inverse agonist of the receptors GPR3, GPR6, and GPR12 and elevates AEA levels [20,28,29].

## 3. The Roles of the Endocannabinoid System in Immunity

It is now clear that many of the components of the endocannabinoid system function as key regulators of the immune system and the immune response. The endocannabinoid system plays an important role in migration of hematopoietic stem and progenitor cells. Kose et al. demonstrated that endocannabinoids can stimulate migration of human hematopoietic stem cells in a cannabinoid receptor dependent manner [30]. They also showed that in healthy individuals, the concentration of the endocannabinoid 2-AG in blood plasma is higher than in bone marrow plasma. Pereira et al. proved that CB2 has a role in the retention of immature B cells in the bone marrow [31] and Hoggatt et al. demonstrated a significant decrease in CXCR4 in bone marrow cells treated with the CB1/CB2 agonist CP55940 [32]. Our own recently published results demonstrate that CB2 is involved in the inhibition of lymphocyte recovery after bone marrow transplantation (BMT) [33].

**Figure 1:**
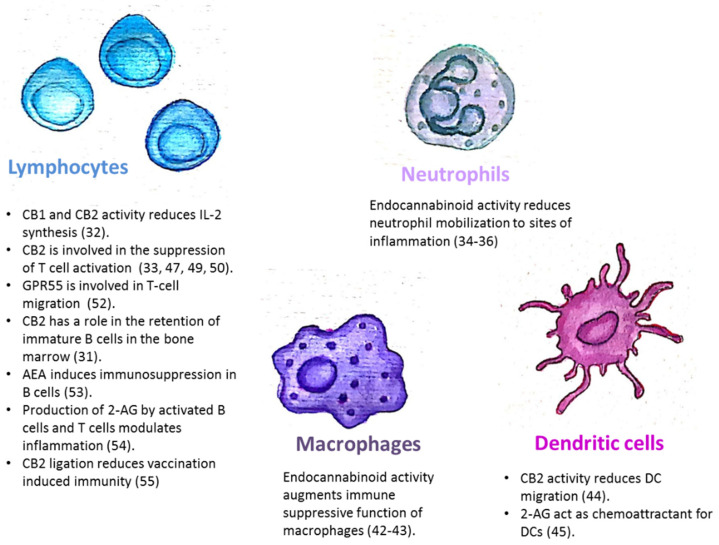
The endocannabinoid system is involved in the regulation of immune cell trafficking and effector cell functions. CB1/CB2: Cannabinoid receptor 1/2; AEA: *N*-arachidonoylethanolamine; 2-AG: 2-arachidonoylglycerol.

The endocannabinoid system is also involved in the regulation of mature immune cell trafficking and effector cell functions (Figure 1). For example, Szabady et al. demonstrated how endocannabinoids play a key regulatory role in the function of intestinal neutrophils. They demonstrated that the transporter P-glycoprotein (P-gp) secretes endocannabinoids into the intestinal lumen that counteract the pro-inflammatory actions of the neutrophil chemoattractant eicosanoid hepoxilin A3. Moreover, the anti-inflammatory actions of P-gp—secreted endocannabinoids—are mediated by CB2 receptors on neutrophils [34]. Kapellos et al. recently published that the CB2 deficiency exacerbates acute neutrophils mobilization to sites of inflammation [35]. In the respiratory syncytial virus (RSV) infection in mice, the level of neutrophil infiltrating the pulmonary airways following an RSV infection was significantly enhanced by blocking CB2 with AM630 [36]. Other innate immune cells are also regulated by endocannabinoids. Both murine and human monocytes/macrophages and microglial cells, express the CB1 and CB2 receptors [16,37,38,39,40,41]. Research by Acharya et al. showed that the engagement of the cannabinoid receptors augments the number and immune suppressive function of the regulatory CX3CR1hi macrophages in the gut [42]. In addition, CB2 is involved in the inhibitory function of tumor associated macrophages [43]. Adhikary et al. demonstrated that signaling through CB2 suppresses dendritic cell (DC) migration by inhibiting the matrix metalloproteinase 9 expression [44]. On the other hand, Maestroni suggested that the endocannabinoid 2-AG may act as a chemotactic substance capable of recruiting DCs and/or their precursors during the innate immune response [45].

The endocannabinoid system is also involved in the regulation of adaptive immunity. Although T-cells express less CB2 than other immune cells, it has been demonstrated that T-cell’s stimulation can upregulate the expression of CB2 receptors [46,47], and also induce CB1 expression [48]. In vitro assays revealed that cannabinoids inhibit T-cell activation via CB2 and other receptors, [33,47,49]. IL2 is a fundamental cytokine involved in T-cell activity and differentiation, secreted from activated T-cells, NK cells, and DCs. Both CB1 and CB2 receptor engagement reduce IL-2 synthesis [50]. In an in vivo model of experimental autoimmune encephalomyelitis, CB2-deficient T-cells in the central nervous system exhibited reduced levels of apoptosis, a higher rate of proliferation and increased production of inflammatory cytokines, resulting in severe clinical disease [51]. Sumida et al. recently demonstrated a role for another receptor, GPR55, in intraepithelial T-lymphocyte migration dynamics in the small intestine [52].

The endocannabinoid system’s role in a mature B-cell function was demonstrated by Eisenstein et al. who showed that anandamide induces dose-related immunosuppression in both the primary and secondary in vitro plaque-forming cell assays of antibody formation [53]. Sido et al. used a delayed-type hypersensitivity in vivo model to demonstrate that the production of 2-AG by activated B-cells and T-cells modulates inflammation [54]. Importantly, Dotsay et al. recently demonstrated that CB2 ligation reduces vaccination-induced immunity. Transient administration of the CB2 antagonists during immunization heightened the intensity and breadth of antigen-specific antibody responses in young and aged mice [55].

Taken together, these examples indicate that the endocannabinoid system is a key regulator of the immune system (Figure 1), therefore any treatment which modulates its function will have immunologic effects.

## 4. The Effects of Cannabinoid-Based Treatments in Different Immune Related Diseases

**Figure 2:**
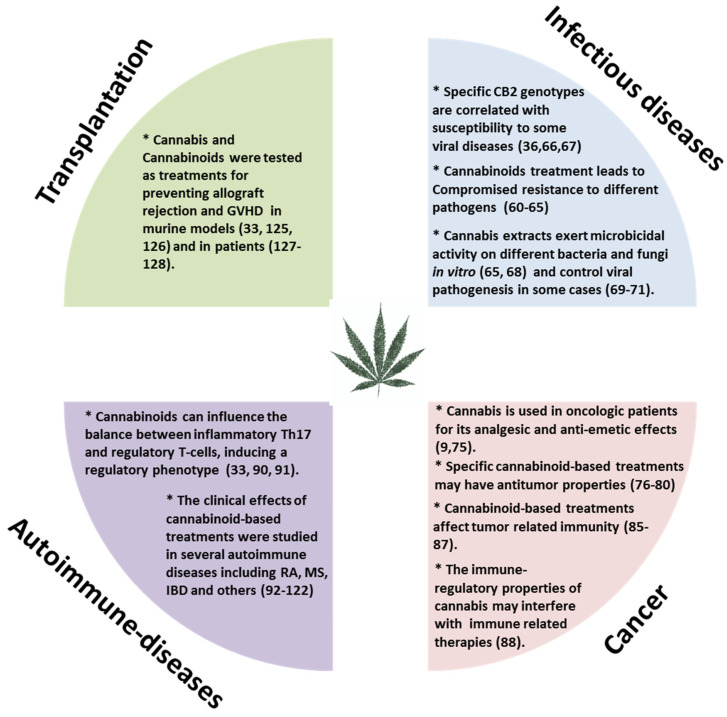
The effects of cannabinoid-based treatments in immune related diseases. GVHD: Graft versus host disease; RA: Rheumatoid arthritis; MS: Multiple sclerosis; IBD: Inflammatory bowel disease; CB2: Cannabinoid receptor 2.

Moderate inflammation provides a beneficial protective effect against infections and long-term adaptive immunity toward specific pathogens. However, chronic or uncontrolled inflammation, resulting from unremitting immune system activation, often causes persistent tissue damage [56]. For example, patients with various autoimmune diseases, undergo treatment to gain control of their immune system and thereby reduce inflammation. On the other hand, in those with suppressed immunity, such as cancer patients we aim to boost the immune response against the tumor. Our research and many other studies have demonstrated immune-regulatory properties for *Cannabis* and cannabinoid-based treatments [33,57,58]. The effects of cannabinoid-based treatments in some immune related diseases (Figure 2) will be reviewed here.

### 4.1. Infectious Diseases

The immune system has the important function of defending the host from foreign agents and pathogens. Researchers have explored the influence of cannabinoids on the immune system’s reaction against pathogens for more than 40 years. In 1977, Bradley et al. showed that the combination of THC and lipopolysaccharide (LPS) is highly toxic in mice and the lethal capability of heat-killed bacteria is enhanced when THC is administered [59]. The same group also investigated the effects of THC and *Cannabis* extracts on host resistance to Listeria, monocytogenes, and herpes simplex virus [60]. Compromised resistance to different pathogens was demonstrated during the following years [61,62,63,64]. Later on, the roles of the endocannabinoid system in the induction of immunity by bacterial pathogens were established [65] and specific CB2 genotypes were found to correlate with susceptibility to some viral diseases [36,66,67]. On the other hand, in vitro studies demonstrated that cannabinoids exert microbicidal activity on different bacteria and fungi [65,68] and could also control viral pathogenesis in some cases [69,70,71]. In a murine model for Malaria, oral administration of *Cannabis* increased the survival of infected mice [72]. In addition, Batugedara et al. observed increased levels of endocannabinoids in the lung and intestine of helminths infected mice and demonstrated that this elevation was associated with improved host immunity [73].

Taken together, the clinical effect of a cannabinoid-based treatment in infectious diseases is the consequence of both its anti-inflammatory effect and its influence on the specific pathogen.

Another subject for consideration is the role of the endocannabinoid system in vaccination and the effects of *Cannabis* and cannabinoid-based treatments on vaccination related immunity. Dotsey et al. investigated the effect of transient CB2 blockade on the immune response to vaccination in young and aged mice and demonstrated that such treatment heightens the intensity and breadth of antigen-specific immune responses [55]. However, a prospective study of humoral and cellular immune responses to hepatitis B vaccination in habitual marijuana smokers did not reveal a major impact on the development of systemic immunity [74].

### 4.2. Cancer

Currently, cannabinoid-based treatments are administered to oncologic patients as palliative medicines due to their analgesic and anti-emetic effects [9,75] (Table 1). Recent studies suggested that some cannabinoid-based treatments might also have antitumor properties [76,77]. However, most of these studies utilized in vitro methods, a few were done in immune-competent animal models [78,79,80], and the data from human patients is anecdotal. In addition, a large diversity was found in the expression of endocannabinoid related molecules in different kinds of tumors [81,82], and therefore cannabinoid-based treatments with dissimilar compositions may be effective only in specific cancer sub-types [77].

The tumor microenvironment is a complex ecosystem, including blood vessels, immune cells, fibroblasts, extracellular matrix, cytokines, hormones and other factors. In addition to the tumor itself, different factors of the tumor microenvironment are involved in cancer progression. It is now clear that malignant development and progression is highly dependent on immune responses [83]. In recent years, immunotherapy has revolutionized the era of cancer treatment, restoring tumor-induced immune deficiency in the tumor microenvironment and modulating immune responses against cancers [84]. The understanding of cannabinoid-based treatments effects on the tumor microenvironment’s immunity is essential to provide personalized therapeutical plans for varying oncologic patients and for the development of future combined therapies. Unfortunately, data from basic and medical research dedicated to this subject is currently limited. McKallip et al. reported that THC enhances breast cancer growth and metastasis by suppression of the antitumor immune response, in mice injected with 4T1 tumor cells [85]. In models of lung carcinoma and alveolar carcinoma, THC treated immune-competent mice demonstrated enhanced tumor growth but not in immune-incompetent mice, indicating an immune-related mechanism [86]. Whereas in a model of melanoma xenograft others reported inhibition of tumor growth by synthetic CB1 or CB2 agonists in both immune-competent mice and immune-incompetent mice [87]. In an ex vivo experiment, Zgair et al. showed that CBD and THC had similar anti-proliferative effects, both on PBMCs isolated from patients on chemotherapy regimens for non-seminomatous germ cell and on PBMCs from healthy volunteers [58].

Only one study has investigated the possible drug-drug effects of cannabinoids with immunotherapy. In retrospective, an observational study in patients treated with nivolumab, Taha et al. demonstrated that the use of *Cannabis* during immunotherapy treatment decreased the response rate, without affecting progression-free survival or overall survival and without relation to the *Cannabis* composition [88].

### 4.3. Autoimmune Diseases

Some of the most established data on the endocannabinoid system’s immune related roles and the effects of phyto- and synthetic- cannabinoids on immunity, come from pre-clinical studies of autoimmune diseases [20,89]. Several groups have shown that cannabinoids can influence the balance between inflammatory Th17 and regulatory T-cells [33,90,91], inducing a regulatory phenotype. The clinical effects of cannabinoid-based treatments were studied in several inflammatory, autoimmune related diseases. Rheumatoid arthritis (RA) is one of the most prevalent autoimmune diseases, statistics show it is among the main causes of disability worldwide, causing unbalanced pain and joint malformation and destruction. Malfait et al. showed that the CBD treatment effectively blocks the progression of arthritis in a murine collagen-induced arthritis model [92]. Similarly, Gui et al. demonstrated that the activation of cannabinoid receptor 2 attenuates synovitis and joint destruction in collagen-induced arthritis in mice [93]. Another interesting approach is elevating endocannabinoid levels by inhibition of their degrading enzymes. Lowin et al. and McDougll et al. demonstrated beneficial effects for treatments that include FAAH inhibitors in murine models for osteoarthritis (OA) and RA [94,95]. In patients with OA and RA increased FAAH activity was detected in the synovia and the endocannabinoids AEA and 2-AG were identified in the synovial fluid [96]. However, although several FAAH inhibitors were entered into clinical trials for different applications, none of them have reached approval for clinical use thus far, [97] and one of the phase I studies was terminated due to neurologic adverse effects in some of the participants [98]. The efficacy, tolerability, and safety of a *Cannabis*-based medicine (Sativex) to relieve pain caused by RA was assessed in one clinical trial [99]. In this randomized, double-blind, parallel group study with 58 patients over five weeks of treatment, a significant analgesic effect was observed and disease activity was significantly suppressed. A study that will test the efficacy and safety of using CBD and THC for the treatment of pain in patients with inflammatory arthritis is planned (Table 1).

Multiple sclerosis (MS) is a progressive, long-term autoimmune demyelinating disease of the central nervous system. Neuroprotection in experimental autoimmune encephalomyelitis by *Cannabis*-based cannabinoids was demonstrated in animal models [51,100,101,102]. Cannabinoid-based treatments were also examined clinically in MS patients. Sexton et al. detected a significant increase in the endocannabinoid AEA, in serum from individuals with MS compared to control subjects. The effect of *Cannabis* use on the tested immunological properties was similar in patients and in control subjects. For example, serum levels of IL-17 were significantly reduced in non-naive subjects, whether cases or controls [103]. Several randomized clinical trials examined the efficacy of cannabinoid treatment in MS. In a meta-analysis of clinical trials on the use of cannabinoids for spasticity due to MS, da Rovare et al. found an insignificant difference related to spasticity between cannabinoids and placebo (*p* = 0.18) [104]. Recently, Akgün et al. systematically reviewed publications on the THC:CBD spray use for refractory MS spasticity and found that the proportion of patients reaching the threshold of minimal clinical important (CI) difference, with at least a 20% reduction of the spasticity Numeric Rating Scale score after four weeks ranged from 41.9% to 82.9% [105]. A current study examines the efficacy of *Cannabis* in spasticity due to MS (Table 1).

Inflammatory bowel disease (IBD) is a general classification based on symptomology. It is comprised of well-known gastrointestinal inflammatory disorders such as Ulcerative colitis (UC), Crohn’s (CD), etc. Cannabinoid receptors as well as endocannabinoids are upregulated in inflammation, and their presence and stimulation attenuates murine colitis, while cannabinoid receptor antagonism and cannabinoid receptor deficient models reverse these anti-inflammatory effects [8,106]. Both FAAH and MAGL inhibitors reduce disease scores in colitis mouse models [107,108]. In patients, some endocannabinoid molecules were differentially expressed in various bowel diseases. For example, AEA and oleoylethanolamide (OEA) were increased in the plasma of UC and CD patients while 2-AG was elevated in patients with CD, but not UC [109].

Several questionnaire-based studies have validated *Cannabis* use in 6.8–15.9% of adult patients with IBD. The most common reasons given were to alleviate abdominal pain, diarrhea, or anorexia [110]. In a small prospective placebo-controlled study, Naftali et al. demonstrated significant clinical, steroid-free benefits for 10 out of 11 patients with an active CD treated with THC rich *Cannabis*; although induction of remission was not achieved within the eight-week study [111]. Irving et al. performed a randomized, double-blind, placebo-controlled, parallel-group, study of cannabidiol-rich botanical extract in the symptomatic treatment of UC. In this study, the physician’s global assessment of illness severity, subject global impression of change, and patient-reported quality-of-life were improved for patients taking a CBD-rich botanical extract (*p* = 0.069, *p* = 0.003, and *p* = 0.065, respectively). However, patients were less tolerant of the CBD-rich botanical extract compared with the placebo [112]. Recently, Mbachi et al. evaluated several UC-related complications and clinical endpoints in a total of 298 *Cannabis* users with UC as compared to a propensity score matched group of nonusers with UC. Their results show that the prevalence of partial or total colectomy was lower in *Cannabis* users compared to nonusers (*p* = 0.01) and there was a trend toward a lower prevalence of bowel obstruction (*p* = 0.057). *Cannabis* users had a shorter duration of hospitalization (*p* < 0.007) compared to their nonuser counterparts [113]. Current studies examine combined THC and CBD drops for the treatment of Crohn’s disease and the use of a synthetic cannabinoid, Nabilone, in IBD patients (Table 1). In addition to their anti-inflammatory effects, the beneficial consequences of the cannabinoids’ treatment in IBD may be attributed by their influence on the permeability of the gastrointestinal tract. Couch et al. recently demonstrated that aspirin causes an increase in the absorption of lactulose and mannitol, which could be reversed by palmitoylethanolamide (PEA) or CBD treatment (*p* < 0.001) in a human, randomized, double-blind, controlled trial [114].

There is also evidence that suggests the involvement of cannabinoids in other autoimmune diseases. For example, Hegde et al. demonstrated attenuation of experimental autoimmune hepatitis by cannabinoids [115]. In a mouse model of myasthenia gravis, a cannabinoid receptor agonist reversed fatiguing failure of neuromuscular transmission [116]. Anti-inflammatory effects were also evident in a rodent model of autoimmune uveoretinitis treated with a CB2-selective agonist [117]. In a murine model for systemic lupus erythematosus (SLE), the CBD treatment was found to accelerate disease progression [118] and in patients, an involvement of the endocannabinoid system in the pathogenesis of this disease was described [119,120]. An ongoing study will examine the efficacy of JBT-101, a CB2 agonist, in SLE patients (Table 1). A decreased likelihood of diabetes for *Cannabis* users versus non-users was also suggested [121,122] and currently the anti-inflammatory properties of *Cannabis* and their relevance to insulin sensitivity in Type 2 diabetes is being examined (Table 1).

### 4.4. Transplantation

Transplantation medicine is a rapidly evolving field and over the last years there has been substantial progress in organ exchange strategies. Allogeneic organ/BMT therapy is dependent on the success of suppressing recipient immune responses to the foreign organ (organ rejection) and/or the pathologic immunity of the transplant against the recipient’s tissues (known as graft versus host disease–GVHD). Immunosuppressive drugs are the standard of care, although they are associated with numerous side effects, among the most significant are opportunistic infections and transplant-related malignancies.

Due to their anti-inflammatory properties, cannabinoids were suggested as potential treatments for preventing allograft rejection/GVHD [123,124]. The efficacy of cannabinoids, and particularly *Cannabis* extract, was demonstrated in murine models of GVHD [33,125] and skin allograft [126]. The efficacy of CBD as a prophylactic treatment for GVHD was also demonstrated in a phase II clinical trial [127]. Cuñetti et al. showed pain improvement in six out of seven kidney transplant patients receiving CBD, but they did not examine the effect on organ rejection [128]. Greenan et al. did not find any significant effect from the *Cannabis* recreational use on kidney allograft outcomes at one-year after renal transplantation. However, in these patients *Cannabis* use was defined by a positive urine toxicology screen and/or self-reported recent use, and the *Cannabis* chemotypes, time of administration, and doses vary [129].

## 5. How to Choose the Best Treatment?

Many cannabinoid-based treatments are currently available; they are either based on pure cannabinoids or on different chemotypes of the *Cannabis* plant. Although there is abundant information regarding the influence of *Cannabis* and cannabinoids on the immune system, the effect of pure cannabinoids such as THC and CBD and different *Cannabis* treatments was rarely compared. Several papers, including our own, demonstrated that *Cannabis* based treatments achieve better clinical results than pure cannabinoids (known as the entourage effect), in animal models [33,130,131]. It has been suggested that this is due to the combination of cannabinoids with other active molecules in the plant, for example terpenes and flavonoids [132].

In order to provide evidence-based data to better understand the medical potential in *Cannabis* treatments, several groups have compared different *Cannabis* chemotypes for the treatment of specific conditions. For example, Kamal et al. used a systems approach for finding *Cannabis* chemotypes with anxiolytic properties [133] and Johnson et al. compared the efficacy, safety, and tolerability of THC+CBD *Cannabis* extracts and the THC *Cannabis* extract in patients with intractable cancer-related pain [134]. In addition, Morgan et al. tested the individual and combined effects of pure THC and CBD on psychotomimetic symptoms and memory function [135]. However, none of these studies have tested the immunological consequences of these treatments. In our murine models, the effects of THC-high *Cannabis* extract and CBD-high *Cannabis* extract on inflammation were not significantly different [33].

The accumulating data about various molecules that can be found in *Cannabis* plants [5] together with advanced laboratory methods, allows for a better characterization of the *Cannabis* chemotypes used for therapy. Hopefully in the future clinicians will be able to prescribe the most efficient chemotype for a specific disease.

## 6. Conclusions

The essential regulatory role that the endocannabinoid system plays in immunity makes it a candidate for medical intervention in cases of immune related diseases. However, since the endocannabinoid system is widely expressed in the body and deeply involved in the function of the neurological system, body metabolism, and bone homeostasis [23], it is extremely important to consider all consequences of the treatment. On the other hand, the immune-modulatory effects of cannabinoid-based medicines may be detrimental in other instances. For example, medications that include cannabinoids such as THC and CBD may be used to treat nausea, chronic pain, sleeping disorders, or epilepsy. However, in some oncologic patients, administration of such medications may hold the risk of suppressing desired immunological reactions against pathogens and tumor cells. To these immunological concerns we should add the risks of *Cannabis* allergies [136,137,138] and cross-allergies, mostly due to its botanical origins [139]. In addition, it is essential to consider that cannabinoid-based medicines may reduce the efficacy of vaccination, mainly in children and elderly patients.

## Figures and Tables

**Table 1:** Selected current clinical trials, synthetic, and botanical cannabinoid-based therapies in immune related diseases.

Indication	Treatment	Bot/syn	Phase	Status (Updated on)	ClinicalTrials.gov Identifiers/EudraCT Number
**Inflammation** (Observational study)	*Cannabis*	bot	n.a.	Recruiting (August 2018)	NCT03522103
Cachexia; **Advanced Cancer**	THC/CBD	syn	Phase 3	Not yet recruiting (May 2020)	NCT04001010
Glioblastoma; **Cancer**	THC/CBD	n.a.	Phase 1-2	Not yet recruiting (April 2020)	NCT03529448
Chemotherapy induced Nausea and Vomiting; **Pancreatic Cancer**	Dronabinol	syn	Phase 3	Recruiting (April 2020)	NCT03984214 2019-000616-28
Chemotherapy induced Peripheral Neuropathy; **Cancer**	Cannabinoids	n.a.	Phase 2	Recruiting (September 2019)	NCT03782402
Cancer cachexia; **Pancreatic cancer**	Cannibinols	syn	n.a.	Ongoing (January 2018)	2017-000530-54
Solid Tumor; **Cancer**	*Cannabis*	bot	n.a.	Recruiting (March 2020)	NCT03617692
**Rheumatoid arthritis**	THC/CBD	bot	n.a.	Temporarily Halted (October 2018)	2017-004226-15
Spasticity Due to **Multiple Sclerosis**	BX-1	bot	Phase 3	Recruiting (January 2020)	NCT03756974
**Inflammatory Bowel Diseases**	Nabilone	syn	n.a.	Not yet recruiting (February 2020)	NCT03422861
**Crohn’s Disease**	THC/CBD	n.a.	Phase 1-2	Completed (March 2019)	NCT01826188
**Systemic Lupus Erythematosus**	JBT-101	syn	Phase 2	Recruiting (March 2020)	NCT03093402
**Type 2 Diabetes**	*Cannabis*	bot	n.a.	Recruiting (March 2020)	NCT04114903

Sources: ClinicalTrials.gov, EU Clinical Trials Registery. Abbreviations: syn: Synthetic, bot: Botanical; n.a.: Not available.
